# Bilateral acute macular neuroretinopathy in a young woman after the first dose of Oxford–AstraZeneca COVID-19 vaccine

**DOI:** 10.1016/j.ajoc.2022.101281

**Published:** 2022-01-20

**Authors:** Pierre-Henry Gabrielle, Florian Baudin, Ines Ben Ghezala, Cyril Meillon, Alain Marie Bron, Louis Arnould, Catherine Creuzot-Garcher

**Affiliations:** a Department of Ophthalmology, Dijon University Hospital, Dijon, France; b Eye and Nutrition Research Group, CSGA, UMR, INRA, 6265 CNRS, Burgundy University, Dijon, France; c Physiopathologie et Épidémiologie Cérébro-Cardiovasculaires, (PEC2, EA 7460), Burgundy University, Dijon, France; d INSERM & Dijon University Hospital, CIC1432, Clinical Epidemiology Unit, Dijon, France

**Keywords:** COVID-19, Vaccination, Acute macular neuroretinopathy, AMN, Side effect

## Abstract

**Purpose:**

To report a case of bilateral acute macular neuroretinopathy following the first dose of Oxford-AstraZeneca COVID-19 (coronavirus disease 2019) vaccine in a young, Caucasian, and healthy woman.

**Observations:**

A 25-year-old Caucasian female patient presented to the ophthalmology department of Dijon University Hospital with a 3-week history of black spots and paracentral scotoma in both eyes. She had no past medical history and was using the combined estrogen-progestin oral contraceptive (COC). These symptoms occurred 24 h after receiving the first Oxford-AstraZeneca COVID-19 vaccination dose. The ophthalmologic signs were preceded a few hours earlier by fever and flu-like symptoms. Ophthalmologic examination revealed a preserved visual acuity with a quiet anterior segment and normal fundus in both eyes. Findings on multimodal retinal imaging, particularly near-infrared reflectance (NIR) and optical coherence tomography (OCT) imaging, were classical of an acute macular neuroretinopathy in both eyes.

**Conclusions and importance:**

COVID-19 vaccination is justified as an essential public health measure. Acute macular neuroretinopathy may occur in patient receiving a COVID-19 vaccination dose. Further reports are needed to confirm this association. Physicians should be aware of this complication and request an eye examination with at least OCT or NIR imaging in the case of any visual symptoms after vaccination, notably in young women using COC.

## 1 Introduction

The Oxford–AstraZeneca coronavirus disease-2019 (COVID-19) vaccine, codenamed “ChAdOx1 nCoV-19” or “AZD1222,” is a chimpanzee adenoviral vectored vaccine with a full-length severe acute respiratory syndrome coronavirus-2 (SARS-CoV-2) spike insert, co-developed at the University of Oxford (Oxford, UK) and AstraZeneca (Cambridge, UK). The interim analysis of ongoing clinical trials published to date showed that AZD1222 has an acceptable safety profile and is efficacious against symptomatic COVID-19.^1^ The vaccine's local and systemic reactogenicity was well tolerated. The side effects were less frequent and of lower intensity in older adults, with lower doses, and after the second dose.^1^ Ocular involvement after vaccination is rare and a few reported cases have been recently published after COVID-19 vaccination.^2^, ^3^, ^4^ The development of new imaging technologies in ophthalmology, such as optical coherence tomography (OCT), has significantly changed the understanding, diagnosis, and management of many ocular diseases, including those resulting from posterior uveitis.^5^

## 2 Case report

Herein, we report a case of bilateral acute macular neuroretinopathy (AMN) after administration of the first dose of the AZD1222 vaccine. A 25-year-old White female patient presented to the ophthalmology department of Dijon University Hospital with a 3-week history of black spots and paracentral scotoma in both eyes. These symptoms occurred 24 h after receiving the first vaccination dose. She did not describe any change in her visual symptoms before our examination. The ophthalmologic signs were preceded a few hours earlier by fever and flu-like symptoms that are common side effects in young people after the AZD1222 vaccine. The patient had no past medical history or allergy. She was using the combined estrogen-progestin oral contraceptive (COC), was a non-smoker, and denied any recent illicit drug use.

**Fig. 1:**
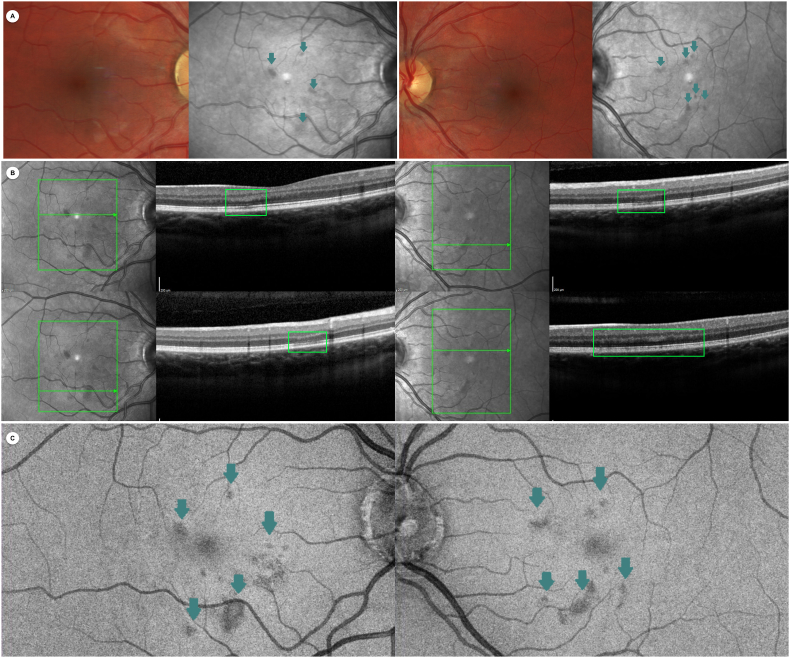
Multimodal retinal imaging findings. The right and left columns represent the right and left eyes, respectively. Panel A (first row): Color fundus photography and near-infrared reflectance (NIR) imaging. NIR imaging shows multiple hyporeflective parafoveal wedge-shaped areas in both eyes (green arrows). Panel B (second row): Combined NIR and optical coherence tomography (OCT) imaging. OCT imaging reveals bilateral multiple localized hyperreflective macular lesions of the outer plexiform layer and Henle layer associated with thinning of the outer nuclear layer, as well as localized disruption of the ellipsoid zone (EZ) and interdigitation zone and an attenuated external limiting membrane (green squares on B-scan OCT). Panel C (third row): En face-OCT imaging at the EZ level showing multiple hyporeflective lesions in both eyes (green arrows). (For interpretation of the references to colour in this figure legend, the reader is referred to the Web version of this article.)

**Fig. 2:**
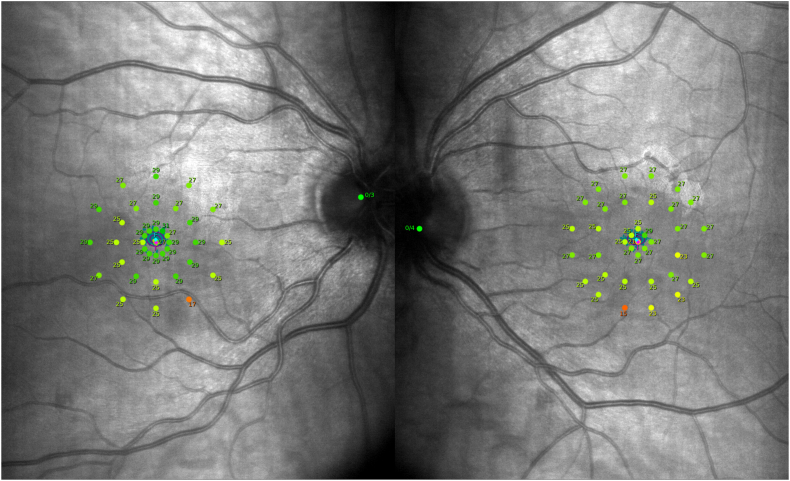
Macular integrity assessment microperimetry of the patient. The right and left columns represent the right and left eyes, respectively. The microperimetry shows multiple reduced macular sensitivity points correlating with the near-infrared reflectance lesions.

On examination, her visual acuity was preserved with 20/20 Snellen acuity in both eyes and anterior segment was normal. Findings on multimodal imaging were classic for AMN and are presented in the Fig. 1.^6^ Color Fundus photography and autofluorescence imaging were unremarkable in both eyes. However, near-infrared reflectance (NIR) imaging showed multiple classic hyporeflective parafoveal wedge-shaped areas (Fig. 1, panel A). B-scan OCT revealed multiple bilateral localized hyperreflective macular lesions of the outer plexiform layer and Henle layer associated with thinning of the outer nuclear layer, as well as localized disruption of the ellipsoid zone (EZ) and interdigitation zone and an attenuated external limiting membrane (Fig. 1, Panel B). En face-OCT at the EZ level showed hyporeflective lesions colocalizing with some NIR imaging abnormalities (Fig. 1, Panel C). Swept-source OCT angiography (Carl Zeiss Meditech, Germany) did not show any vascular abnormalities in either eye, notably in the deep retinal capillary plexus, choriocapillaris and choroid. Visual fields were normal, although macular integrity assessment microperimetry (CenterVue, Padova, Italy) showed multiple reduced macular sensitivity points correlating with NIR and OCT lesions (Fig. 2). Dye fundus angiography was unremarkable with normal transit and no sign of posterior uveitis. A full laboratory work-up was normal, including complete blood count, C-reactive protein, comprehensive metabolic panel, and viscosity and coagulability panel. COVID-19, syphilis, Bartonella, tuberculosis, and HIV test results were also negative.

## 3 Discussion

AMN is a rare disorder of the outer retinal layers that preferentially affects healthy young Caucasian women and is commonly associated with non-specific flu-like illness, fever, or oral contraceptive use.^5^, ^6^, ^7^ It can affect one or both eyes with symptoms ranging from paracentral scotomas to impaired visual acuity. Fortunately, its prognosis is usually favorable within weeks with an improvement of symptoms and lesions without any specific treatment. Cases of AMN after COVID vaccination and influenza vaccination were recently reported and strengthen a possible association between AMN and vaccination.^5^^,^^8^, ^9^, ^10^, ^11^, ^12^ Although the pathogenesis of AMN remains uncertain and complex, a retinal microvascular etiology affecting the deep retinal capillary plexus (DCP) is suggested.^6^^,^^8^ It is plausible that relative hypovolemia associated with fever and flu-like illness post-vaccination or possibly subclinical small deep retinal capillary vasculitis leading to ischemia of the DCP. A transient inflammatory process induced by the vaccination could have led to outer retinal alterations. Furthermore, choroidal layer involvement have been previously reported to be a possible pathogenetic mechanism in AMN, although choroidal circulation segmentation flow in OCT-angiography was normal in our case.^13^ COC, a risk factor for AMN, may also stress this hypoperfusion due to the thrombotic risk, as in our case and notably in the case of young female smokers. Recently, a few cases of a rare clotting disorder have been linked to AZD1222 and described as vaccine-induced prothrombotic immune thrombocytopenia.^14^^,^^15^ This seems unlikely in our case, since the blood count (platelet count: 210,000 platelets/mL) and coagulability profile were within normal. The onset of the first symptoms was 24 h after the vaccination, whereas in VIPIT this occurs at least 4 days after vaccination.

## 4 Conclusion

COVID-19 vaccination is justified as an essential public health measure, and all authorized vaccines have been proven to be safe and efficacious. Our case adds to other reports of side effects with potential serious post-immunization complications, although AMN has a favorable visual prognosis in most cases. Further reports are needed to confirm this association. Physicians should be aware of this complication and request an eye examination with at least OCT or NIR imaging in the case of any visual symptoms after vaccination, notably in young women using oral contraceptive.

## 5 Patient consent

The patient's written consent for publication was obtained.

## Institutional review board

Due to its non-interventional character and retrospective design, the Medical Ethics Committee of the Dijon University Hospital approved the use of this case report data.

## Funding

None.

## Authorships

All authors provided care for the patient. PHG and FB drafted the manuscript. FB, LA, IBG, CM, AMB and CCG provided supervision, discussion, and additional suggestions. All authors attest that they meet the current ICMJE criteria for Authorship.

## Declaration of competing interest

We declare no competing interests.
